# Risk factors and impact on outcomes of high-density shadow on immediate cerebral CT after successful interventional recanalization in acute ischemic stroke with large vessel occlusion

**DOI:** 10.3389/fneur.2025.1663994

**Published:** 2025-11-21

**Authors:** Wensheng Zhang, Weifang Xing, Jiyun Feng, Yangchun Wen, Minzhen Zhu, Haiping Lan, Xiaojing Zhong, Zhenqin Jiang, Li Ling

**Affiliations:** 1 Department of Neurology, Heyuan People’s Hospital, Guangdong Provincial People's Hospital Heyuan Hospital, Heyuan, Guangdong, China; 2 Department of Neurology, Shenzhen Hospital, Southern Medical University, Shenzhen, Guangdong, China; 3 Shenzhen School of Clinical Medicine, Southern Medical University, Shenzhen, Guangdong, China; 4 Heyuan Key Laboratory of Molecular Diagnosis & Disease Prevention and Treatment, Doctors Station of Guangdong Province, Heyuan People's Hospital, Heyuan, Guangdong, China; 5 Department of Neurology, Lianzhou People's Hospital, Lianzhou, Guangdong, China

**Keywords:** large vessel occlusion, acute ischemic stroke, interventional recanalization, high-density shadow, immediate cerebral CT

## Abstract

**Objective:**

To explore the risk factors, classification, relation with hemorrhage and clinical significance of high-density shadow on immediate cerebral CT in patients with large vessel occlusion acute ischemic stroke after successful interventional recanalization.

**Methods:**

A retrospective analysis was conducted on patients with acute ischemic stroke due to anterior circulation large vessel occlusion who received interventional recanalization from January 2019 to December 2023 in Heyuan People’s Hospital. The main inclusion criteria included NIHSS score ≥ 6 points at the time of onset, the time from onset to femoral artery puncture ≤ 24 h and so on. The main exclusion criteria included pre onset mRS score > 2 points, the vital signs were unstable during the onset of the disease and so on. Variables we studied included NIHSS score at admission, preoperative ASPECT score, blood flow reperfusion eTICI grading, surgical methods and so on. According to the distribution, density, volume, etc. of cerebral hyperdensity, high-density shadow was divided into cortical type, soft type, metallic type, and diffuse type.

**Results:**

318 patients showed high-density shadow on cerebral CT immediately after successful interventional recanalization. In multiple logistic regression analysis, the history of hypertension and preoperative ASPECT score were correlated independently with the occurrence of high-density shadow. 27 patients experienced symptomatic intracranial hemorrhage. It was found that high-density shadow was not independent with symptomatic intracranial hemorrhage in univariate logistic regression analysis (*p* > 0.05). In the classification of high-density shadow, there were 16 cases of cortical type, 85 cases of soft type, 80 cases of metallic type, and 137 cases of diffuse type. Patients with diffuse type had the highest incidence of futile recanalization, symptomatic intracranial hemorrhage, malignant brain edema, and highest mortality rate within 3 months after surgery (*p* < 0.05).

**Conclusion:**

A low preoperative ASPECT score was an independent risk factor of high-density shadow on immediate cerebral CT after successful interventional recanalization, while a history of hypertension, mere use of balloon angioplasty and combination of balloon angioplasty and stent implantation may serve as a protective factor. Patients with diffuse high-density shadow had the worst prognosis and the highest incidence of symptomatic intracranial hemorrhage and malignant brain edema.

## 1 Introduction

Acute ischemic stroke with large vessel occlusion has a high incidence rate, mortality and disability rate (1). Interventional recanalization has been proven to be effective and safe in the treatment of acute ischemic stroke caused by large vessel occlusion (2–9). However, high-density shadow on immediate cerebral computed tomography (CT) after interventional recanalization is a common imaging manifestation, with an incidence rate of up to 30–65% (10, 11). However, current researches on high-density shadow on immediate cerebral CT after interventional recanalization is relatively limited, and the essence of high-density shadow may be contrast agent enhancement, contrast agent exudation, hemorrhage, or a combination of contrast agent enhancement, exudation, and hemorrhage. At present, there is a lack of unified understanding of the clinical significance of high-density shadow, and there is no research exploring independent risk factors of high-density shadow (12–14). If the interpretation of the meaning of high-density shadow is incorrect, such as judging the contrast agent as hemorrhage, it will delay antithrombotic treatment. Exploring the clinical significance of immediately postoperative cerebral CT high-density shadow is of great significance. Our study aimed to explore the independent risk factors of high-density shadow on immediate cerebral CT after successful interventional recanalization of acute ischemic stroke with anterior circulation large vessel occlusion, relation with hemorrhage and the impact of high-density shadow on patients’ prognosis and complications. At the same time, referring to a previous research on arterial thrombolysis, we attempted to propose an improved classification of high-density shadow on immediate cerebral CT after successful interventional recanalization, and explored the differences in prognosis and complications among different types of high-density shadow of patients.

## 2 Materials and methods

### 2.1 Study population

Data of patients with acute ischemic stroke due to anterior circulation large vessel occlusion and received successful interventional recanalization in our single comprehensive stroke center from January 2019 to December 2023 was retrospectively collected. This research plan had been approved by the Medical Ethics Committee of Heyuan People’s Hospital (YXYJLL-YJZFQ33). Our study complied with the Declaration of Helsinki. Because this study only collected clinical data and conducted retrospective analysis without any form of intervention on the treatment plan of patients, the Medical Ethics Committee approved the exemption of patients’ informed consent in this study.

**Figure 1:**
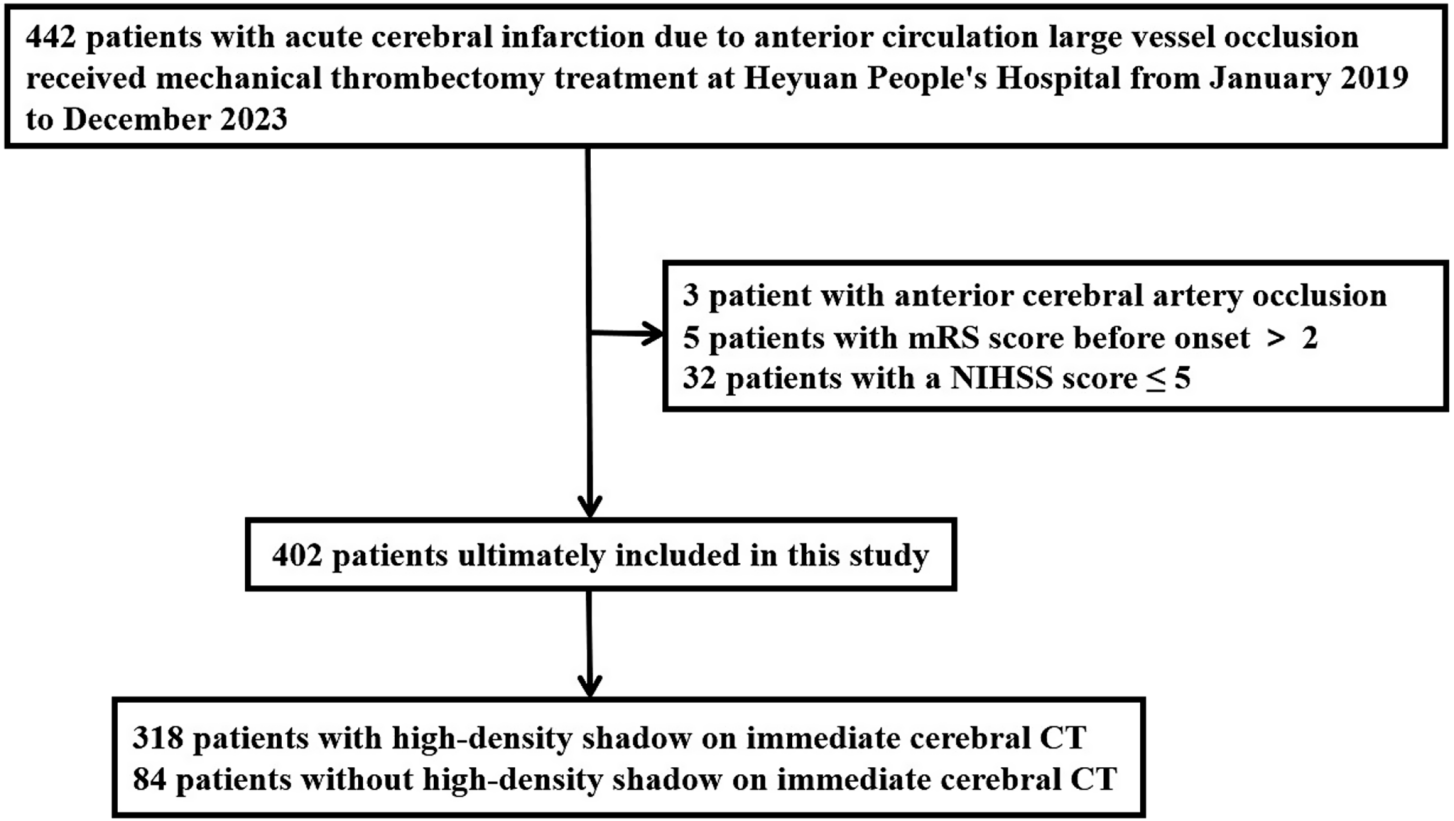
Research flowchart. mRS, modified Rankin Scale; NIHSS, National Institute of Health stroke scale; CT, computed tomography.

Inclusion criteria for this study: (1) It was confirmed occlusion of major blood vessels in the anterior circulation by preoperative imaging examination, including the internal carotid artery, M1 segment of the middle cerebral artery, and M2 segment of the middle cerebral artery; (2) National Institute of Health stroke scale (NIHSS) score ≥ 6 points at the time of onset; (3) Preoperative Alberta Stroke Program Early Computed Tomography (ASPECT) score ≥ 6 points; (4) Age ≥ 18 years old; (5) Pre onset modified Rankin Scale (mRS) score ≤ 2 points; (6) The time from onset to femoral artery puncture ≤ 24 h; (7) The patient’s family members signed and agreed to undergo interventional recanalization treatment; (8) Received immediate postoperative CT scan. Exclusion criteria: (1) Acute ischemic stroke with posterior circulation large vessel occlusion; (2) Occlusion of anterior cerebral artery; (3) Pre onset mRS score > 2 points; (4) The vital signs were unstable during the onset of the disease. The modified Rankin Scale is a scale used to evaluate the neurological recovery status of stroke patients, with a score range of 0 to 6 points. The higher the score, the worse the prognosis, point of 0 represents no symptoms at all, and point of 6 represents death. The research flowchart is shown in Figure 1.

### 2.2 Patient and public involvement

Patients or the public WERE NOT involved in the design, or conduct, or reporting, or dissemination plans of our research.

### 2.3 Methods of interventional recanalization and postoperative management

After imaging evaluation, it was confirmed that the acute ischemic stroke was caused by occlusion of large blood vessels in the anterior circulation, and there was a salvageable ischemic penumbra. After the patient’s family members signed and agreed to perform interventional recanalization, the treatment was carried out under local anesthesia and sedation. If the patient’s condition required, endotracheal intubation and general anesthesia can be administered. The interventional physicians selected one or more methods of stent thrombectomy, thrombus aspiration, balloon dilation, stent implantation, etc. for interventional recanalization based on the patient’s lesion location, nature, and vascular pathway during the operation. The thrombectomy stents we used included Solitaire AB, RECO, TREVO, the suction catheters we used included Sofia, ACE Penumbra, and Tianxun distal access catheter, the balloon we used was Gateway balloon dilation catheter, the carotid stent we used was Protege stent, and the intracranial stent we used was Apollo intracranial vascular stent. In terms of postoperative management, each group of patients would be monitored and their blood pressure and blood sugar would be controlled according to the latest guidelines, and received at least 24 h of electrocardiogram monitoring to closely monitor their vital signs. After surgery, appropriate antithrombotic drugs would be selected based on the patient’s pathogenesis, and the appropriate timing for initiating antithrombotic drugs would be used to treat the patient with antithrombotic therapy. Early postoperative prevention of deep vein thrombosis would be routinely performed on patients. According to the presence or absence of high-density shadows on the immediate postoperative cerebral CT, we divided the patients into a group with high-density shadow and a group without high-density shadow.

### 2.4 Classification method for high-density shadow

**Figure 2:**
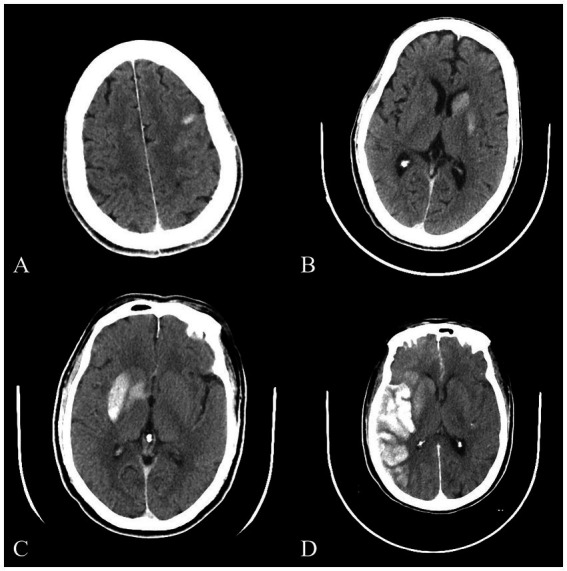
Typical images of four types of high-density shadow on immediate cerebral CT after successful interventional recanalization. **(A)** Cortical high-density shadow; **(B)** Soft high-density shadow; **(C)** Metal high-density shadow; **(D)** Diffuse high-density shadow. CT, computed tomography.

After successful interventional recanalization, immediate cerebral CT examination should be performed to determine whether the patient had high-density shadow. Referring to a previous classification method for high-density shadow of immediate cerebral CT after arterial thrombolysis in patients with acute ischemic stroke (15), we had made some improvements to it. Based on the distribution, density, volume, and other information of high-density shadow, we had classified them into four types: (1) Cortical high-density shadow: focal high-density shadow limited to the cortex without occupying space effect. (2) Soft high-density shadow: a high-density shadow limited to the brain parenchyma, with a density of less than 80 HU, a volume of less than one-third of the cerebral hemisphere, no occupying effect, and no high-density shadows in the sulci, gyrus, or ventricles. (3) Metal high-density shadow: a high-density shadow confined to the brain parenchyma, with a density greater than or equal to 80 HU, a large volume but less than one-third of the cerebral hemisphere, no high-density shadow in the sulci, gyrus, or ventricles, and may be accompanied by mild space occupying effect. (4) Diffuse high-density shadow: the high-density shadow has a large volume and may not be limited to the brain parenchyma. It may be accompanied by obvious occupying effects and may be accompanied by high-density shadow in the sulci, gyrus, and ventricles. The typical cases of high-density shadow in four types of immediate cerebral CT are shown in Figure 2. The classification of high-density shadow on immediate CT after successful interventional recanalization was evaluated by two imaging experts who were unaware of the patient’s condition. When there was a disagreement in the evaluation, the third expert should conduct the evaluation. When three experts had different opinions, a final consensus would be reached through thorough discussion.

### 2.5 Data collection

Demographic, medical history, NIHSS score, preoperative ASPECT score, collateral circulation score, surgical process characteristics, and other information of all patients were collected. The American Society of Interventional and Therapeutic Neuroradiology/ Society of Interventional Radiology (ASTIN/SIR) scoring system was used to score collateral circulation (16), with a score of 0: no collateral blood flow reached the ischemic area. 1 point: There is slow collateral blood flow reaching the ischemic peripheral area. 2 points: There is rapid collateral blood flow reaching the ischemic peripheral area, but only a portion reaches the ischemic area. 3 points: Slow but complete blood flow can be seen throughout the entire ischemic area in the late venous stage. 4 points: Through retrograde perfusion, blood flow rapidly and completely perfuses the entire ischemic area. Symptomatic intracranial hemorrhage is defined as bleeding visible on the patient’s cerebral CT scan, and an increase of ≥ 4 points in the patient’s NIHSS score (17, 18). The definition of futile recanalization is that the mRS score of the patient is greater than 2 points 3 months after successful interventional recanalization, and no independent neurological function has been achieved (19, 20). The characteristics of the surgical process included the time from femoral artery puncture to recanalization of occluded blood vessels, and the time from onset to recanalization of occluded blood vessels. The extended thrombolysis in cerebral infarction (eTICI) blood flow grading is used to evaluate the recanalization of occluded vessels. Levels 0 to 2a are defined as failed intervention recanalization, while levels 2b to 3 are defined as successful intervention recanalization.

### 2.6 Statistical analysis

All statistical analyses were conducted using SPSS 26.0 software. Measurement data was expressed as mean ± standard deviation or median (interquartile range). Statistical analysis was performed using t-test for measurement data that conformed to normal distribution, and rank sum test was used for measurement data that did not conform to normal distribution. Count data was expressed in frequency and percentage, and statistical analysis was performed using chi square test. Factors found to be associated with high-density shadow on immediate cerebral CT after successful interventional recanalization in univariate analysis were included into univariate and multivariate logistic regression analysis to determine independent related factors.

## 3 Results

### 3.1 Clinical baseline data of patients after successful interventional recanalization

The average age of all patients was 66.89 ± 11.69 years, with 271 males (64.71%). At the onset of the disease, the NIHSS score was 12.00, the preoperative ASPECT score was 7.00, the collateral circulation score was 3.00, and the time from femoral artery puncture to recanalization of occluded blood vessels was 95.00 min. All patients underwent a 3-month follow-up after surgery.

We included a total of 402 patients, according to whether there was a high-density shadow on the immediate cerebral CT after successful interventional recanalization, the patients were divided into a group with high-density shadow and a group without high-density shadow, with 318 cases (79.10%) in the high-density shadow group and 84 cases (20.90%) in the without high-density shadow group. There were statistically significant differences (*p* < 0.05) in hypertension, preoperative ASPECT score, collateral circulation score, surgical methods and time from femoral artery puncture to recanalization of occluded vessels between the two groups of patients. Compared with the group without high-density shadow, patients in high-density shadow group had a lower rate of history of hypertension, lower preoperative ASPECT scores, lower proportion of mere use of balloon angioplasty, lower proportion of combination of balloon angioplasty and stent implantation and longer time from femoral artery puncture to recanalization of occluded vessels. There was no statistical difference between the two groups in age, sex, history of diabetes, history of coronary heart disease, history of atrial fibrillation, history of cerebral infarction, history of smoking, history of drinking, pre onset mRS score, rate of receiving intravenous thrombolysis treatment, location of occluded vessels, time from onset to recanalization of occluded vessels, and pathogenesis (*p* > 0.05). The baseline clinical data of two groups of patients with and without high-density shadow on immediate cerebral CT after successful interventional recanalization are shown in Table 1.

**Table 1:** Comparison of baseline clinical data between patients with and without high-density shadow on immediate cerebral CT after successful interventional recanalization.

Variables	All patients (*n* = 402)	With high-density shadow (*n* = 318)	Without high-density shadow (*n* = 84)	*p* value
Age, years, (mean ± standard deviation)	66.89 ± 11.69	66.83 ± 12.07	67.10 ± 10.17	0.974
Gender, male, *n* (%)	271 (67.41)	208 (65.41)	63 (75.00)	0.095
Medical history				
Hypertension, *n* (%)	217 (53.98)	162 (50.94)	55 (65.48)	**0.017**
Type 2 diabetes, *n* (%)	73 (18.16)	56 (17.61)	17 (20.24)	0.578
Coronary heart disease, *n* (%)	49 (12.19)	40 (12.58)	9 (10.71)	0.642
Atrial fibrillation, *n* (%)	76 (18.91)	64 (20.13)	12 (14.29)	0.224
Cerebral infarction, *n* (%)	63 (15.67)	52 (16.35)	11 (13.10)	0.465
Smoking history, *n* (%)	161 (40.05)	126 (39.62)	35 (41.67)	0.734
Drinking history, *n* (%)	92 (22.89)	70 (22.01)	22 (26.19)	0.418
Pre onset mRS score, (mean ± standard deviation)	0.08 ± 0.35		0.07 ± 0.34	0.611
The situation at the onset of stroke				
NIHSS score at admission, median (IQR)	12.00 (10.00–16.00)	12.00 (10.00–15.00)	12.00 (8.25–16.00)	0.454
Preoperative ASPECT score, median (IQR)	7.00 (7.00–8.00)	7.00 (7.00–8.00)	8.00 (7.00–9.00)	**<0.001**
Received intravenous thrombolysis treatment, *n* (%)	144 (35.82)	118 (37.11)	26 (30.95)	0.295
Location of occluded blood vessels, *n* (%)				0.082
M1 segment of middle cerebral artery	195 (48.51)	153 (48.11)	42 (50.00)	
M2 segment of middle cerebral artery	22 (5.47)	18 (5.66)	4 (4.76)	
Internal carotid artery	56 (13.93)	38 (11.95)	18 (21.43)	
Anterior circulation tandem lesion	129 (32.09)	109 (34.28)	20 (23.81)	
Collateral circulation score, median (IQR)	3.00 (2.00–3.00)	2.00 (2.00–3.00)	3.00 (2.00–3.00)	**0.001**
Blood flow reperfusion eTICI grading, *n* (%)				**0.008**
<2b	93 (23.13)	83 (26.10)	10 (11.90)	
2b	24 (5.97)	21 (6.60)	3 (3.57)	
2c-3	285 (70.90)	214 (67.30)	71 (84.52)	
Surgical methods				<0.001
Mere use of stent retriever	74 (18.41)	64 (20.13)	10 (11.90)	
Mere use of contact aspiration	7719.15()	62 (19.50)	15 (17.86)	
Combination of stent retriever and contact aspiration	96 (23.88)	82 (25.79)	14 (16.67)	
Mere use of balloon angioplasty	12 (2.99)	5 (1.57)	7 (8.33)	
Combination of balloon angioplasty and stent implantation	23 (5.72)	13 (4.09)	10 (11.90)	
Balloon angioplasty and/or stent implantation after stent retriever and/or contact aspiration	120 (29.85)	92 (28.93)	28 (33.33)	
Time node				
Time from onset to recanalization of occluded blood vessels, min, median (IQR)	543.50 (410.75–760.25)	547.50 (422.00–745.75)	503.50 (354.25–891.25)	0.763
Time from femoral artery puncture to recanalization of occluded vessels, min, median (IQR)	95.00 (73.00–127.00)	98.00 (76.50–128.25)	81.50 (60.25–116.50)	**0.004**
Pathogenesis				0.137
Large artery atherosclerosis	189 (47.01)	143 (44.97)	46 (54.76)	
Cardiogenic embolism	115 (28.61)	98 (30.82)	17 (20.24)	
Other mechanisms	98 (24.38)	77 (24.21)	21 (25.00)	

CT, computed tomography; mRS, modified Rankin Scale; NIHSS, National Institute of Health stroke scale; ASPECT, Alberta Stroke Program Early Computed Tomography; eTICI, extended thrombolysis in cerebral infarction.

When the *p* value is less than 0.05, the corresponding *p* value is bolded, indicating statistical significance.

### 3.2 Univariate and multivariate logistic regression analysis of factors related to high-density shadow on immediate cerebral CT after successful interventional recanalization

The indicators with statistical differences in chi square test, t-test, and non parametric test were included in univariate and multivariate logistic regression analysis. After adjusting for confounding factors, it was found that the history of hypertension (OR = 0.479; 95% CI 0.283–0.810; *p* = 0.006), preoperative ASPECT score (OR = 0.748; 95% CI 0.571–0.979; *p* = 0.034), mere use of balloon angioplasty (OR = 0.150; 95% CI 0.038–0.602; *p* = 0.007) and combination of balloon angioplasty and stent implantation (OR = 0.216; 95% CI 0.070–0.663; p = 0.007) were independently correlated with high-density shadow on immediate cerebral CT after successful interventional recanalization in patients with acute anterior circulation cerebral infarction caused by large vessel occlusion. The univariate and multivariate logistic regression analyses are shown in Table 2.

**Table 2:** Univariate and multivariate regression analysis of high-density shadow on immediate cerebral CT after successful interventional recanalization.

Variables	Univariate analysis			Multivariate analysis		
	OR	95%CI	*p* value	OR	95%CI	*p* value
Hypertension	0.548	0.332–0.903	**0.018**	0.492	0.284–0.852	**0.011**
Preoperative ASPECT score	0.668	0.541–0.824	**<0.001**	0.743	0.563–0.981	**0.036**
Collateral circulation score	0.583	0.425–0.799	**0.001**	0.823	0.550–1.231	0.343
Blood flow reperfusion eTICI grading						
<2b	Reference			Reference		
2b	0.843	0.213–3.340	0.808	0.694	0.168–2.867	0.614
2c-3	0.363	0.179–0.738	**0.005**	0.545	0.253–1.172	0.120
Surgical methods						
Mere use of stent retriever	Reference			Reference		
Mere use of contact aspiration	0.646	0.270–1.546	0.326	0.687	0.269–1.757	0.433
Combination of stent retriever and contact aspiration	0.915	0.382–2.195	0.843	0.638	0.253–1.610	0.341
Mere use of balloon angioplasty	0.112	0.030–0.421	**0.001**	0.150	0.038–0.602	**0.007**
Combination of balloon angioplasty and stent implantation	0.203	0.070–0.586	**0.003**	0.216	0.070–0.663	**0.007**
Balloon angioplasty and/or stent implantation after stent retriever and/or contact aspiration	0.513	0.233–1.131	0.098	0.485	0.209–1.123	0.091
Time from femoral artery puncture to recanalization of occluded vessels	1.009	1.002–1.015	**0.007**	1.007	0.999–1.015	0.085

CT, computed tomography; ASPECT, Alberta Stroke Program Early Computed Tomography; eTICI, extended thrombolysis in cerebral infarction.

When the *p* value is less than 0.05, the corresponding *p* value is bolded, indicating statistical significance.

### 3.3 Comparison of prognosis and complications between patients with and without high-density shadow on immediate cerebral CT after successful interventional recanalization

**Figure 3:**
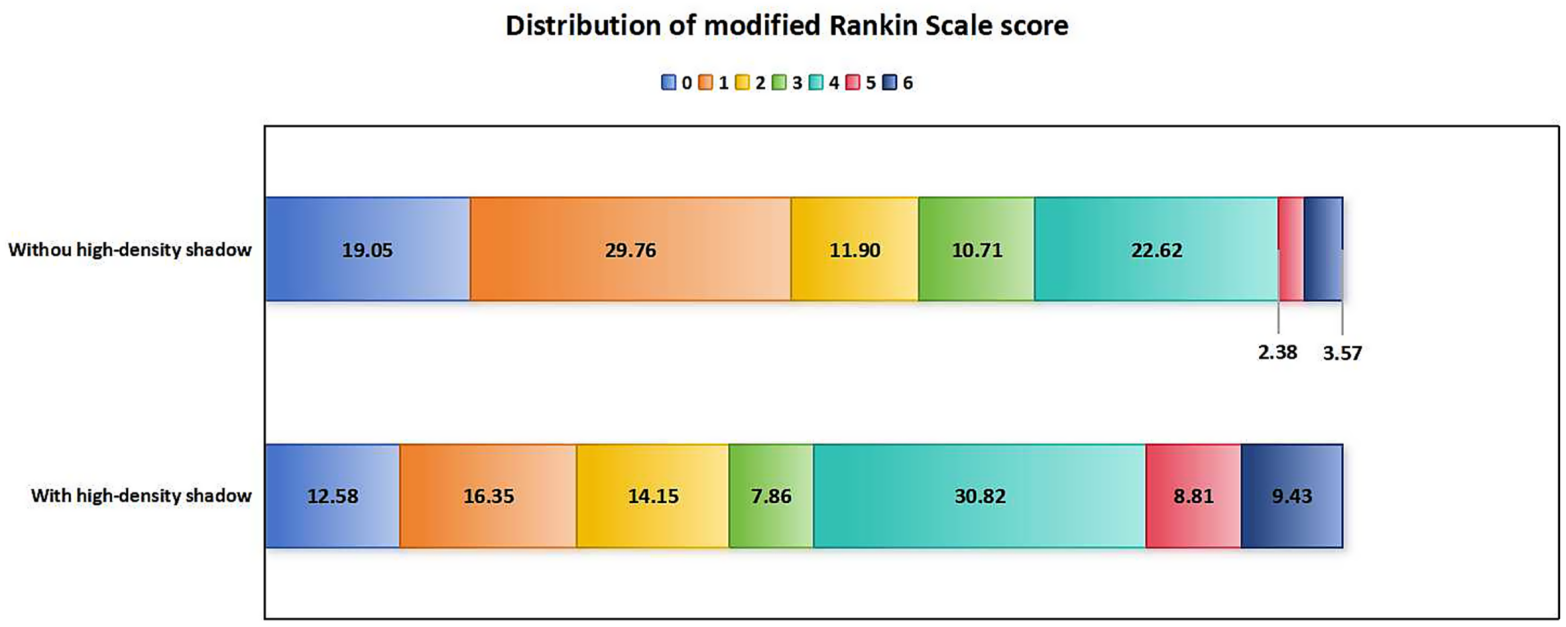
Distribution of mRS scores in patients with and without high-density shadow on immediate cerebral CT 3 months after successful interventional recanalization. mRS, modified Rankin Scale; CT, computed tomography.

In terms of prognosis and complications, compared with patients without high-density shadow, patients in the high-density shadow group had higher mRS scores, higher incidence of futile recanalization, symptomatic intracranial hemorrhage and malignant brain edema, higher mortality rate within 3 months after surgery, and a lower proportion of mRS ≤ 1 at 3 months after surgery (*p* < 0.05). The comparison of prognosis and complications between the two groups of patients is shown in Table 3. The distribution of mRS scores between the two groups of patients 3 months after surgery is shown in Figure 3.

**Table 3:** Comparison of prognosis and complications between patients with and without high-density shadow on immediate cerebral CT after successful interventional recanalization.

Variables	All patients (*n* = 402)	With high-density shadow (*n* = 318)	Without high-density shadow (*n* = 84)	*p* value
mRS score at 3 months after surgery, median, *n* (%)				**0.009**
0	56 (13.93)	40 (12.58)	16 (19.05)	
1	77 (19.15)	52 (16.35)	25 (29.76)	
2	55 (13.68)	45 (14.15)	10 (11.90)	
3	34 (8.46)	25 (7.86)	9 (10.71)	
4	117 (29.10)	98 (30.82)	19 (22.62)	
5	30 (7.46)	28 (8.81)	2 (2.38)	
6	33 (8.21)	30 (9.43)	3 (3.57)	
Excellent prognosis (3-month mRS ≤ 1 point), *n* (%)	133 (33.08)	92 (28.93)	41 (48.81)	**0.001**
Futile recanalization (3 months mRS ≤ 2 points), *n* (%)	214 (53.23)	181 (56.92)	33 (39.29)	**0.004**
Symptomatic intracranial hemorrhage, *n* (%)	27 (6.72)	26 (8.18)	1 (1.19)	**0.023**
Malignant brain edema, *n* (%)	88 (21.89)	84 (26.42)	4 (4.76)	**<0.001**
Mortality 3 months after surgery, *n* (%)	33 (82.09)	30 (9.43)	3 (3.57)	0.082

CT, computed tomography; mRS, modified Rankin Scale.

When the *p* value is less than 0.05, the corresponding *p* value is bolded, indicating statistical significance.

### 3.4 Baseline clinical data of four types of high-density shadow on immediate cerebral CT after successful interventional recanalization

The high-density shadow on immediate cerebral CT after successful interventional recanalization were divided into four types: cortical type in 16 cases (5.03%), soft type in 85 cases (26.73%), metallic type in 80 cases (25.16%), and diffuse type in 137 cases (43.08%). There were statistically significant differences (*p* < 0.05) in NIHSS score at the time of onset, preoperative ASPECT score, and collateral circulation score among the four types of high-density shadow of patients. There was no significant difference in age, sex, hypertension, diabetes, coronary heart disease, atrial fibrillation, cerebral infarction, smoking, drinking, pre onset mRS score, rate of receiving intravenous thrombolysis treatment, location of occluded vessels, time from onset to recanalization of occluded vessels, time from femoral artery puncture to recanalization of occluded vessels, and pathogenesis among four types of high-density shadow of patients (*p* > 0.05). The baseline clinical data of four types of high-density shadow of patients are shown in Table 4.

**Table 4:** Comparison of baseline clinical data in patients of four types of high-density shadow on immediate cerebral CT after successful interventional recanalization.

Variables	Cortical type (*n* = 16)	Soft type (*n* = 85)	Metallic type (*n* = 80)	Diffuse type (*n* = 137)	*p* value
Age, years, (mean ± standard deviation)	66.44 ± 12.55	66.04 ± 12.85	67.81 ± 11.40	66.80 ± 12.00	0.868
Gender, male, *n* (%)	11 (68.75)	58 (68.24)	52 (65.00)	87 (63.50)	0.895
Medical history					
Hypertension, *n* (%)	11 (68.75)	38 (44.71)	41 (51.25)	72 (52.55)	0.321
Type 2 diabetes, *n* (%)	4 (25.00)	13 (15.29)	12 (15.00)	27 (19.71)	0.635
Coronary heart disease, *n* (%)	3 (18.75)	7 (8.24)	8 (10.00)	22 (16.06)	0.261
Atrial fibrillation, *n* (%)	3 (18.75)	15 (17.65)	12 (15.00)	34 (24.82)	0.317
Cerebral infarction, *n* (%)	4 (25.00)	16 (18.82)	11 (13.75)	21 (15.33)	0.625
Smoking history, *n* (%)	5 (31.25)	39 (45.88)	28 (35.00)	54 (39.42)	0.461
Drinking history, *n* (%)	3 (18.75)	17 (20.00)	17 (21.25)	33 (24.09)	0.880
Pre onset mRS score, (mean ± standard deviation)	0	0.07 ± 0.30	0.10 ± 0.38	0.09 ± 0.38	0.725
The situation at the onset of stroke					
NIHSS score at admission, median (IQR)	8.00 (7.00–12.75)	12.00 (10.00–14.00)	12.00 (10.00–15.75)	13.00 (11.00–16.00)	**0.001**
Preoperative ASPECT score, median (IQR)	9.00 (8.00–9.00)	8.00 (7.00–9.00)	8.00 (7.00–9.00)	7.00 (6.00–7.00)	**<0.001**
Received intravenous thrombolysis treatment, *n* (%)	2 (12.50)	34 (40.00)	31 (38.75)	51 (37.23)	0.208
Location of occluded blood vessels, *n* (%)					**0.002**
M1 segment of middle cerebral artery	7 (43.75)	52 (61.18)	40 (50.00)	54 (39.42)	
M2 segment of middle cerebral artery	1 (6.25)	3 (3.53)	2 (2.50)	12 (8.76)	
Internal carotid artery	5 (31.25)	13 (15.29)	8 (10.00)	12 (8.76)	
Anterior circulation tandem lesion	3 (18.75)	17 (20.00)	30 (37.50)	59 (43.07)	
Collateral circulation score, median (IQR)	3.00 (3.00–3.75)	3.00 (2.00–3.00)	3.00 (2.00–3.00)	2.00 (2.00–2.00)	**<0.001**
Blood flow reperfusion eTICI grading, *n* (%)					0.128
<2b	1 (6.25)	18 (21.18)	24 (30.00)	40 (29.20)	
2b	0 (0)	6 (7.06)	3 (3.75)	12 (8.76)	
2c	15 (93.75)	61 (71.76)	53 (66.25)	85 (62.04)	
Time node					
Time from onset to recanalization of occluded blood vessels, min, median (IQR)	506.00 (361.00–1036.75)	563.00 (454.00–858.50)	554.50 (446.25–714.75)	525.00 (404.00–671.00)	0.222
Time from femoral artery puncture to recanalization of occluded vessels, min, median (IQR)	83.50 (72.50–99.00)	91.00 (61.00–120.50)	95.00 (74.25–125.50)	107.00 (84.00–140.00)	**0.001**
Pathogenesis					0.254
Large artery atherosclerosis	7 (43.75)	42 (49.41)	41 (51.25)	53 (38.69)	
Cardiogenic embolism	3 (18.75)	21 (24.71)	24 (30.00)	50 (36.50)	
Other mechanisms	6 (37.50)	22 (25.88)	15 (18.75)	34 (24.82)	

CT, computed tomography; mRS, modified Rankin Scale; NIHSS, National Institute of Health stroke scale; ASPECT, Alberta Stroke Program Early Computed Tomography; eTICI, extended thrombolysis in cerebral infarction.

When the *p* value is less than 0.05, the corresponding *p* value is bolded, indicating statistical significance.

### 3.5 Comparison of prognosis and complications of four types of high-density shadow of patients on immediate cerebral CT after interventional recanalization

**Figure 4:**
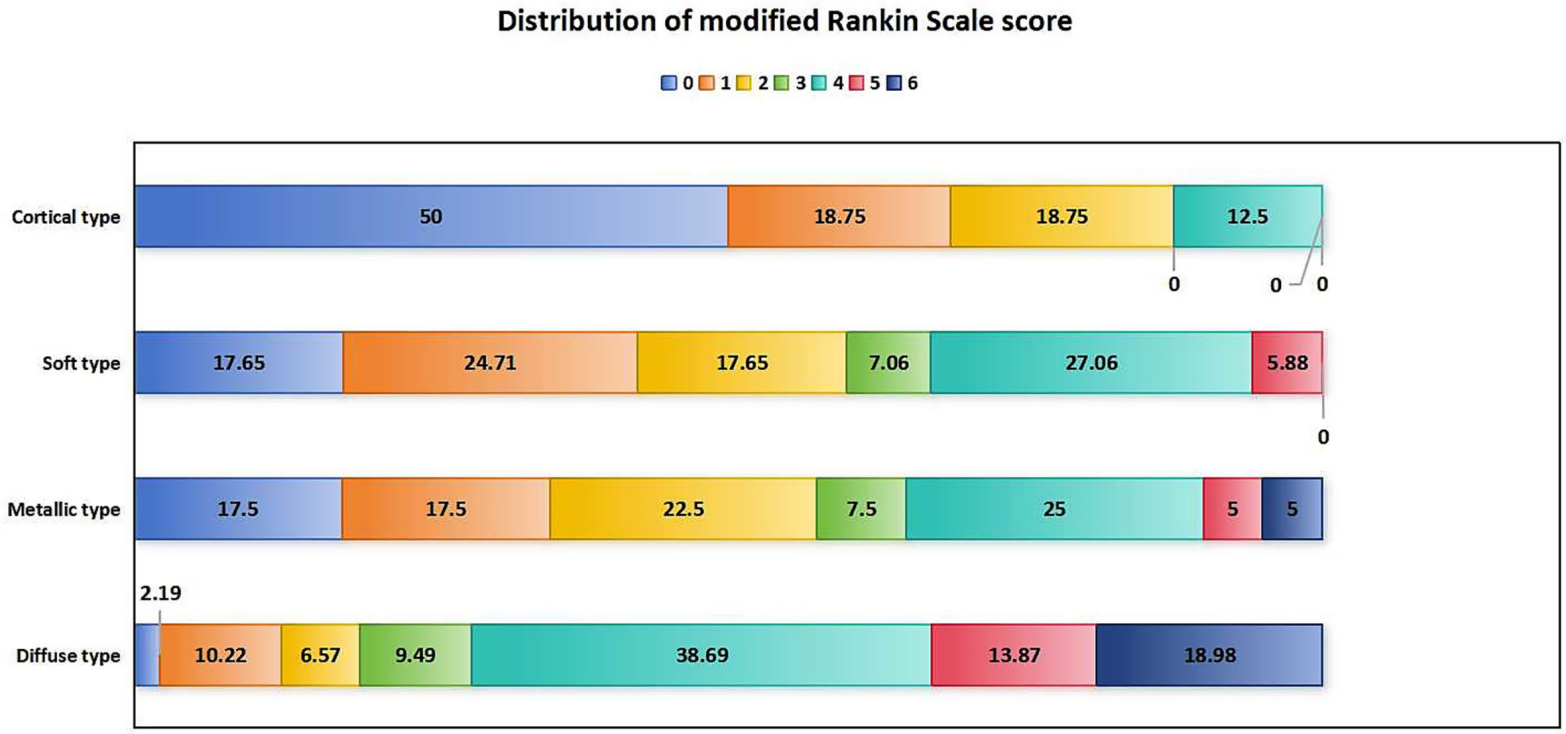
Distribution of mRS scores in patients with four types of high-density shadow on immediate cerebral CT 3 months after successful interventional recanalization. mRS: modified Rankin Scale; CT, computed tomography.

In terms of prognosis and complications, patients with diffuse high-density shadow had the highest mRS score, highest incidence of futile recanalization, symptomatic intracranial hemorrhage and malignant brain edema, highest mortality rate within 3 months after surgery, and lowest proportion of mRS ≤ 1 was at 3 months after surgery (*p* < 0.05). The mRS score, incidence of futile recanalization, symptomatic intracranial hemorrhage and malignant brain edema were the lowest and proportion of mRS ≤ 1 was the highest in patients with cortical high-density shadow at 3 months after surgery (p < 0.05). The comparison of prognosis and complications of four types of high-density shadow of patients are shown in Table 5. The distribution of mRS scores of four types of high-density shadow of patients at 3 months after surgery is shown in Figure 4.

**Table 5:** Comparison of prognosis and complications among patients of four types of high-density shadow on immediate cerebral CT after successful interventional recanalization.

Variables	Cortical type (*n* = 16)	Soft type (*n* = 85)	Metallic type (*n* = 80)	Diffuse type (*n* = 137)	*p* value
mRS score at 3 months after surgery, median (IQR)	0.50 (0–2.00)	2.00 (1.00–4.00)	2.00 (1.00–4.00)	4.00 (3.00–5.00)	**<0.001**
Excellent prognosis (3-month mRS ≤ 1 point), *n* (%)	11 (68.75)	36 (42.35)	28 (35.00)	17 (12.41)	**<0.001**
Futile recanalization (3 months mRS ≤ 2 points), *n* (%)	2 (12.50)	34 (40.00)	34 (42.50)	111 (81.02)	**<0.001**
Symptomatic intracranial hemorrhage, *n* (%)	0 (0)	1 (1.18)	3 (3.75)	22 (16.06)	**<0.001**
Malignant brain edema, *n* (%)	0 (0)	5 (5.88)	9 (11.25)	70 (51.09)	**<0.001**
Mortality 3 months after surgery, *n* (%)	0 (0)	0 (0)	4 (5.00)	26 (18.98)	**<0.001**

CT, computed tomography; mRS, modified Rankin Scale.

When the *p* value is less than 0.05, the corresponding *p* value is bolded, indicating statistical significance.

## 4 Discussion

Our study found that the incidence of high-density shadow on immediate cerebral CT after successful interventional recanalization in patients with acute ischemic stroke due to anterior circulation large vessel occlusion was 79.10%. A low preoperative ASPECT score was an independent risk factor of high-density shadow on immediate cerebral CT after successful interventional recanalization of acute ischemic stroke due to anterior circulation large vessel occlusion, while a history of hypertension, mere use of balloon angioplasty and combination of balloon angioplasty and stent implantation may serve as a protective factor. Among the four types of high-density shadow, patients with diffuse high-density shadow had the worst prognosis, the highest incidence of symptomatic intracranial hemorrhage and malignant brain edema. Our research had a certain degree of innovation by proposing a classification method for high-density shadow in cerebral CT different from ECASS or the PCHD system which were used for classification of cerebral hemorrhage and analyzing the risk factors for the occurrence of high-density shadow, and had the potential to be widely applied in clinical practice of detailed postoperative management and improvement of patient prognosis after interventional recanalization surgery for acute ischemic stroke with large vessel occlusion.

A study by Parrilla et al. (10) explored the clinical significance of high-density shadow on immediate cerebral CT in patients with acute ischemic stroke caused by large vessel occlusion after interventional recanalization. The study found that the incidence of high-density shadow on immediate cerebral CT after interventional recanalization was 31.25%, but it did not increase the risk of symptomatic intracranial hemorrhage or poor prognosis. A study by Shao et al. (12) explored the impact of immediate high-density shadow on immediate cerebral CT on hemorrhage transformation and clinical prognosis in patients with acute ischemic stroke due to large vessel occlusion after interventional recanalization. The incidence of high-density shadow was 57.14%. According to the ECASS classification of hemorrhage transformation and other related studies (21–23), the researchers classified postoperative high-density shadow on immediate cerebral CT into four types: PCHD-1, PCHD-2, PCHD-3, and PCHD-4. It was found that the incidence of hemorrhage transformation in patients without high-density shadow on immediate cerebral CT after surgery was 14.8%, and the incidence of hemorrhage transformation in patients with high-density shadow was 77.8%. The occurrence of high-density shadow on immediate cerebral CT after interventional recanalization of PCHD-3 and PCHD-4 was an early risk indicator for hemorrhage transformation. PCHD-1 was a strong predictor of early deterioration of neurological function, and was associated with relatively good prognosis. In different studies, there might be significant differences in the incidence of high-density shadow on immediate cerebral CT after interventional recanalization, and the correlation between high-density shadow and patients’ hemorrhage transformation and prognosis also varied. It is necessary to further study the incidence, classification and impact on outcomes of high-density shadow on immediate cerebral CT after successful interventional recanalization.

Jang et al. (15) conducted a study exploring the outcome of high-density shadow on immediate cerebral CT after intra-arterial thrombolysis in patients with acute ischemic stroke. The incidence of high-density shadow was 32.98%. According to the volume, shape, location, and density of the high-density shadow, they were divided into four types: cortical type, soft type, metallic type, and diffuse type. It was found that all metallic high-density shadow would lead to hemorrhage transformation, with 50% of diffuse high-density shadow showing hemorrhage transformation, and most soft high-density shadow being benign. Although all metal high-density shadow will experience hemorrhage transformation, some of these hemorrhage transformations were benign. Our research found that the incidence of high-density shadow on immediate cerebral CT after successful interventional recanalization was 79.10%, which was significantly higher than that of patients undergoing arterial thrombolysis. Our study once again confirmed the high incidence of high-density shadow on immediate cerebral CT after interventional recanalization surgery, suggesting that we need to focus on and explore the characteristics and clinical significance of this imaging examination, so that it can play a guiding role in postoperative management. Referring to Jang et al.’s (15) research on the classification method of high-density shadow on immediate cerebral CT, our study made appropriate improvements, adding content such as whether the high-density shadow area was accompanied by space occupying effect and whether it was accompanied by high-density shadow in the sulci, gyrus, and ventricles. We also classified the high-density shadow on immediate cerebral CT after successful interventional recanalization in patients with acute ischemic stroke due to large vessel occlusion into the above four types, making them more suitable for evaluation after interventional recanalization. Among the four types of high-density shadows we modification, patients with diffuse high-density shadow had the worst prognosis, the highest incidence of symptomatic intracranial hemorrhage and malignant brain edema, while patients with cortical high-density shadow had the best prognosis and the lowest incidence of symptomatic intracranial hemorrhage and malignant brain edema. In clinical practice, we had found that patients with metallic and diffuse high-density shadows are more prone to experience symptomatic intracranial hemorrhage, and the contrast agent imaging and bleeding in cerebral CT can overlap. When hemorrhage occurs, it is often accompanied by contrast agent imaging, but when contrast agent imaging occurs, it may not necessarily be accompanied by hemorrhage. Our findings suggested the need to strengthen postoperative management for patients with diffuse high-density shadow after successful interventional recanalization, take targeted measures to reduce the occurrence of complications, and improve patient prognosis.

At present, there is no research exploring the independent related factors of high-density shadow on immediate cerebral CT after successful interventional recanalization. The existing researches on high-density shadows on immediate CT after interventional recanalization mainly focused on the correlation between high-density shadows and hemorrhage transformation, as well as patient prognosis. There is a lack of direct exploration of the risk factors for the occurrence of high-density shadows (10, 12, 14). Further research is needed to investigate the dynamic changes in high-density shadows of cerebral CT at different time points after interventional recanalization surgery and their correlation with hemorrhage transformation and patient prognosis. Our study did not explore the dynamic changes of high-density shadows. Our focus was on the characteristics of high-density shadows of immediate cerebral CT after surgery, and they were classified into four types based on their characteristics. Our study found that a low preoperative ASPECT score was an independent risk factor of high-density shadow on immediate cerebral CT after successful interventional recanalization in patients with acute ischemic stroke due to anterior circulation large vessel occlusion, and a history of hypertension may be a protective factor. The lower the preoperative ASPECT score, the larger the infarct volume, the more amount of location, the more severe the blood–brain barrier damage, and the higher the probability of high-density shadow. However, patients with hypertension history were less likely to have high-density shadow than those without hypertension history. The reason was still unclear. It may be related to the pathogenesis of hypertension patients who are more likely to have large atherosclerosis, and most of the pathogenesis characteristics of this kind of pathogenesis are gradually aggravating, and the infarct size is relatively smaller than that of patients with cardiogenic embolism (24). A prospective study by Kobayashi et al. (25) on carotid endarterectomy found that intentionally increasing blood pressure during surgery can increase the average blood flow velocity of the middle cerebral artery, which can prevent new ischemic lesions caused by microemboli during surgery. In a study by Rots et al. (26) exploring the correlation between hemodynamic instability and surgical stroke during carotid endarterectomy, it was found that preoperative hypertension and significant decrease in intraoperative blood pressure were associated with perioperative cerebral ischemia. A higher blood pressure during surgery could be beneficial for patients because it can increase blood supply to the infarcted area through collateral circulation, so as to reduce the infarct volume. However, this speculation needs to be further incorporated into more cases for verification, and this conclusion needs to be carefully interpreted. Furthermore, we founded that mere use of balloon angioplasty and combination of balloon angioplasty and stent implantation may be a protective factor of high-density shadow, for which the most likely reason was that there are fewer steps involved in mere use of balloon angioplasty and combination of balloon angioplasty and stent implantation, the instrument provided less stimulation to the vascular wall and intima, and the amount of contrast agent used was relatively small.

Our study had certain limitations. Firstly, this was a single center, retrospective study with a relatively limited number of included cases. Secondly, this study only included patients who had received successful interventional recanalization and did not include patients who had failed interventional recanalization. Thirdly, this study only explored the high-density shadow on immediate cerebral CT after successful interventional recanalization, and did not investigate the dynamic evolution characteristics of high-density shadow on cerebral CT after interventional recanalization. Fourthly, we did not include indicators such as hyperlipidemia, presence of bleeding tendency, previous drug history in the baseline clinical analysis. A larger sample size multicenter retrospective or prospective clinical study is needed to confirm our findings.

## 5 Conclusion

A low preoperative ASPECT score was an independent risk factor of high-density shadow on immediate cerebral CT after successful interventional recanalization of acute ischemic stroke due to anterior circulation large vessel occlusion, while a history of hypertension, mere use of balloon angioplasty and combination of balloon angioplasty and stent implantation may serve as a protective factor. Not all high-density shadows are equal, patients with diffuse high-density shadow had the worst prognosis and the highest incidence of symptomatic intracranial hemorrhage and malignant brain edema.

## Data Availability

The raw data supporting the conclusions of this article will be made available by the authors, without undue reservation.
